# Using Surface-Enhanced Raman Spectroscopy to Probe Surface-Localized Nonthermal Plasma Activation

**DOI:** 10.1021/acs.jpclett.4c00747

**Published:** 2024-04-09

**Authors:** Minseok Kim, Lorenzo Mangolini

**Affiliations:** † Department of Mechanical Engineering, University of California, Riverside, Riverside, California 92521, United States; ‡ Materials Science & Engineering Program, University of California, Riverside, Riverside, California 92521, United States

## Abstract

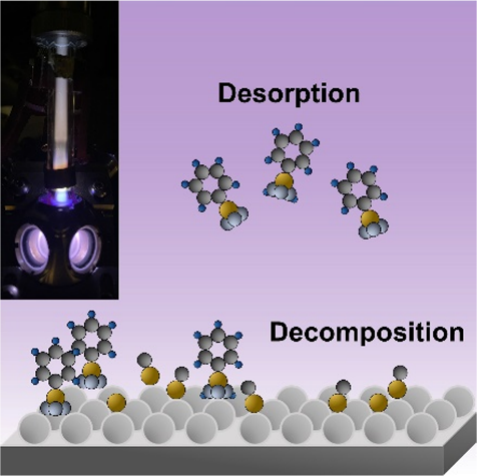

Low-temperature, nonthermal plasmas generate a complex environment even when operated in nonreactive gases. Plasma-produced species impinge on exposed surfaces, and their thermalization is highly localized at the surface. Here we present a Raman thermometry approach to quantifying the resulting degree of surface heating. A nanostructured silver substrate is used to enhance the Raman signal and make it easily distinguishable from the background radiation from the plasma. Phenyl phosphonic acid is used as a molecular probe. Even under moderate plasma power and density, we measure a significant degree of vibrational excitation for the phenyl group, corresponding to an increase in surface temperature of ∼80 °C at a plasma density of 2 × 10^10^ cm^–3^. This work confirms that surface-localized thermal effects can be quantified in low-temperature plasma processes. Their characterization is needed to improve our understanding of the plasma-induced activation of surface reactions, which is highly relevant for a broad range of plasma-driven processes.

Nonthermal plasmas, coupled with heterogeneous catalysis, provide unique pathways for activating a variety of surface reactions.^1−3^ The presence of energetic electrons with typical temperatures in the 1–5 eV range leads to the continuous generation of ions, radicals, metastables, and various excited species even at room temperature. These systems are well known to activate even stable molecules such as N_2_, CH_4_, and CO_2_.^4^ Leveraging these plasma-induced excited species at the surface of catalysts while using electrical power from renewable energy resources has the potential to alleviate environmental concerns associated with conventional thermal processes relying on fossil fuels.^5−10^ Kim et al. reported that vibrationally excited CO_2_ in the nonthermal plasma reacts with the H-terminated Pd surface, significantly reducing the activation barrier for CO_2_ hydrogenation.^11^ Vibrational excitation of N_2_ in nonthermal plasmas is also proposed as a critical mechanism for nitrogen fixation, serving as a counterpart for the Haber-Bosch process.^12,13^ While excitation of molecules in nonthermal plasmas in the gas phase is essential to achieve the desired gas conversion, one overlooked fact is that significant excitation may be present at plasma-exposed surfaces. This can significantly alter the kinetics of surface reactions involving various adsorbates. Unfortunately, quantitative measurements of surface excitation under plasma exposure have remained poorly characterized experimentally. With this letter, we aim to address this issue by utilizing surface-enhanced Raman spectroscopy (SERS) while a nonthermal plasma is impinging on a monolayer of a molecular adsorbate, in this case, phenyl phosphonic acid (PPA).

There is substantial evidence suggesting that the recombination of plasma-produced species at surfaces has profound effects on the processing capabilities of these systems. For instance, Wagner et al. recently pointed out that the recombination of plasma-induced atomic hydrogen at the surface of Mg nanoparticles serves as the primary source of surface heating and actually hinders the full hydrogenation of the Mg particles because of the competing thermal decomposition of MgH_2_.^14^ Similarly, Lopez et al. have shown, via in situ FTIR measurements, that hydrogen rapidly desorbs from silicon nanoparticles immersed in a nonthermal plasma because of rapid thermal desorption.^15^ A few studies describe attempts to quantify these surface-specific effects. Walton et al. used time-domain thermo-reflectance to quantify the plasma-induced energy flux to the surface.^16^ Our group performed Raman thermometry on a few layers of graphene, under exposure of an argon–hydrogen plasma, and found the surface temperature to significantly exceed the bulk material temperature.^17^ One limitation of this approach is the thickness of the graphene sample (∼10 layers), which makes this measurement, strictly speaking, not surface-selective.

Here we perform in situ Raman and FTIR spectroscopy measurements, under plasma exposure, while utilizing a molecular tracer adsorbed onto the plasma-exposed surface, therefore overcoming the limitations of our previous report. We choose phenyl phosphonic acid (PPA) as a molecular tracer due to its strong response in Raman and FTIR.^18−20^ We adsorb PPA onto superthin alumina films (∼2 nm) coated on top of nanostructured silver to enhance the Raman response.^21,22^ We find that even at low RF input power (∼0.5 W) the vibrational temperature of PPA exceeds the substrate temperature by ∼80 °C in the presence of argon plasma. We carried out probe measurements to estimate the plasma density. These measurements reveal a nearly linear relationship between the temperature of PPA and the plasma density, confirming that surface heating scales with the increased flux of plasma-produced species on the surface. For further investigation, we conducted in situ temperature-dependent FTIR measurements with the same argon plasma exposure. We find that the signal from PPA starts to decrease at ∼80 °C lower temperature when exposed to the argon plasma compared to the case without plasma exposure, which is consistent with the findings from the Raman study. Overall, this work presents a widely applicable method for the quantitative analysis of plasma surface interactions through easy-to-access Raman spectroscopy. It confirms that exposure to low-temperature nonthermal plasmas can significantly alter surface reaction kinetics and provides a pathway toward better interpreting plasma-induced reaction pathways.

We have performed in situ Raman spectroscopy to measure the temperature of PPA adsorbed on alumina. Figure 1a describes a laboratory-built Raman setup coupled with a commercial environmental chamber (HVC-DRP-5, Harrick Scientific). The chamber has been modified by replacing one of the KBr windows with a Pyrex tubular reactor. A copper electrode wrapped around the Pyrex tube is connected to a radio frequency (RF) power supply (RFPP RF-5S, Advanced Energy) to ignite the discharge. We maintain a 20 sccm flow rate of argon and a pressure of 3.7 Torr for all experiments. We set the temperature of the substrates using a temperature controller (ATK-024-3, Harrick Scientific). Figure 1b shows the argon plasma in operation. The optical system consists of a continuous-wave laser at λ = 532 nm with a power of up to 1 W. The scattered photons are collected using a visible–near-infrared monochromator (Acton Spectra Pro, Princeton Instruments) and captured by a CCD camera mounted to the exit slit of the monochromator. Details of these measurements are summarized in our previous report and in Methods in the SI.^17^ One of the challenges for these measurements is obtaining a sufficient Raman signal to extract the temperature of PPA in the presence of plasma. The emission from the plasma itself is strong with many emission lines over a broad spectral range.^23^ To address this issue, we have fabricated a surface-enhanced Raman spectroscopy (SERS) substrate to enhance the Raman signal and clearly distinguish it from the background emission from the plasma.^21^ As shown in Figure 1c, a 3-nm-thick Ag film is deposited by e-beam evaporation onto a silicon wafer, which then undergoes a dewetting process to form an island structure (Figure 1d and e). To adsorb PPA onto the surface of catalysts, the dewetted Ag nanoparticles are coated with 2-nm-thick alumina (see Method in the SI). Figure 1f presents a peak for Si only when PPA is applied to the bare Si wafer (black line), small peaks for PPA without nanostructured silver (blue line), and a significantly enhanced Raman signal for PPA when the SERS substrate is used (green line). The calculated enhancement factor (i.e., *I*_with Ag_/*I*_wihout Ag_) is 12, which is relatively lower than the commonly reported SERS enhancement factors. It should be noted that our approach entails the use of a simple SERS substrate through island-structured Ag. There is therefore significant room for improvement in sensitivity by utilizing more sophisticated SERS substrates.^17^

**Figure 1:**
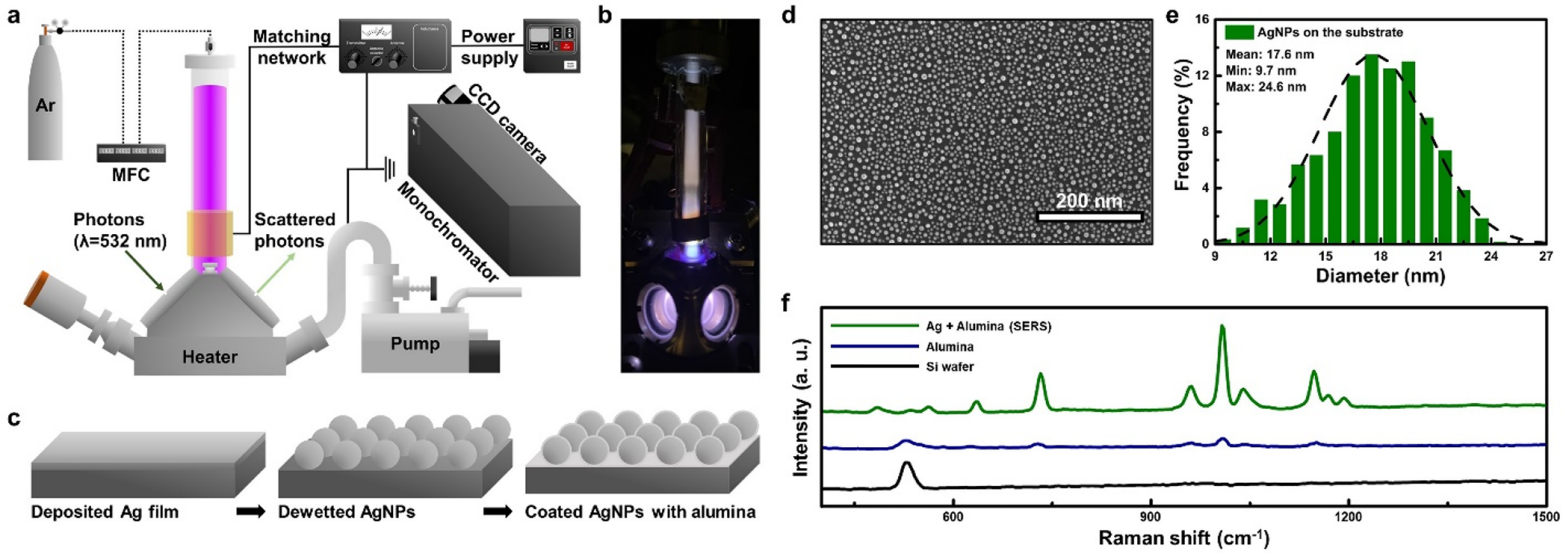
(a) Schematic of the Raman spectroscopy setup coupled with an RF plasma reactor. (b) Photograph of the Ar plasma in operation. One of the KBr windows in the reaction chamber is replaced with a Pyrex tubular reactor to expose the surface to argon plasma. (c) Schematic of the three steps to fabricate nanostructured Ag coated with alumina. (d) SEM image right after the dewetting process of the Ag film. (e) Size distribution analysis of island-structured Ag nanoparticles on the Si wafer. (f) Raman spectra for PPA on a Si water, on alumina-coated Si, and on the SERS substrate. The green line demonstrates significant enhancements compared to both the bare Si wafer and the case without nanostructured Ag.

The Raman spectra for PPA over 1 h confirms the stable chemisorption of PPA on the alumina surface at 3.7 Torr and room temperature (Supporting Information Figure 1). Figure 2a presents the Stokes and anti-Stokes peaks for PPA on alumina using the SERS substrate. Peaks corresponding to ±1001 cm^–1^ and ±719 cm^–1^ represent the C–C–C stretch (aromatic benzene ring) and the P–C stretch of PPA, respectively.^18^ The temperature of the adsorbates is calculated based on the temperature-dependent populations of the vibrational mode.^17^ The ratio of intensity between anti-Stokes and Stokes signals (i.e., *I*_anti-Stoeks_/*I*_Stokes_) is proportional to the temperature of the adsorbates because the thermal population of vibrational modes increases with temperature. Maher et al. studied rhodamine 6G through SERS and reported that while the Stokes peak remained constant regardless of temperature, there was a significant reduction in the anti-Stokes peak when the temperature decreased from 300 K to 170 K.^24^ We have deconvoluted the peaks and fitted them to a Boltzmann peak shape to obtain areas of Stokes and anti-Stokes corresponding to the aromatic ring, as shown in the Figure 2a upper panel. The Raman spectrum (Figure 2b,c) is corrected by subtracting the spectrum with only argon plasma exposure (i.e., without a laser). This is necessary because the argon plasma itself emits photons over a broad wavelength range (Figure 2).

**Figure 2:**
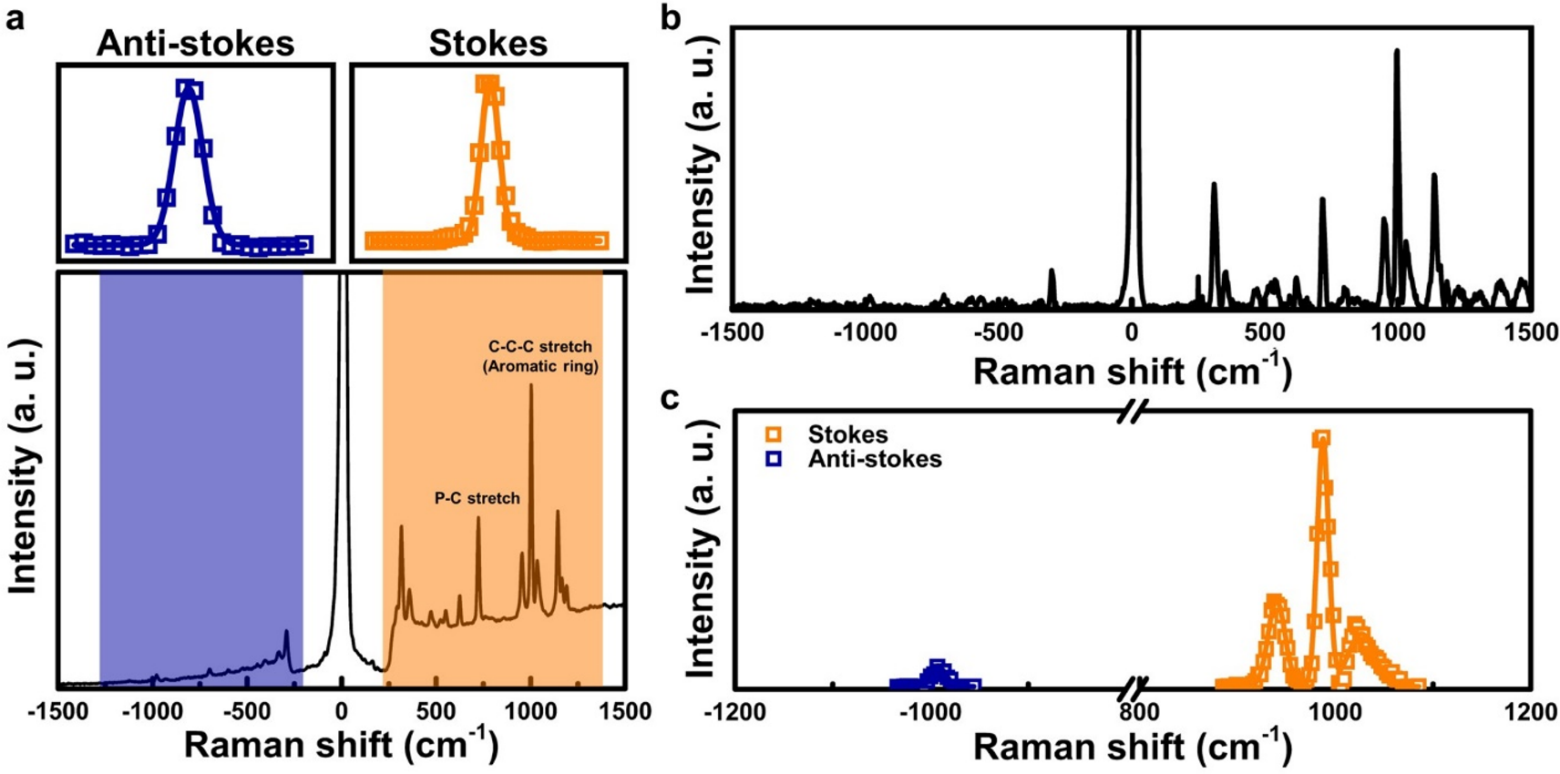
(a) Raman spectrum of PPA absorbed onto the SERS substrate in the absence of argon plasma (square points, measured spectrum; lines, Gaussian fit). (b) Raman spectrum obtained with argon plasma exposure. (c) Gaussian fit to calculate areas of Stokes and anti-Stokes for the aromatic ring of PPA.

Supporting Information Figure 3 shows that the measured temperature using the peak at 1000 cm^–1^, corresponding to the aromatic benzene ring of PPA, highly exceeds the substrate temperature regardless of the laser power, even without a plasma. The use of Raman thermometry, in conjunction with SERS substrates, is complicated by the fact that the Raman enhancement is strongly wavelength-dependent and also affected by the exact placement of the probe molecule with respect to the plasmonic antenna.^25^ It is therefore to be expected that the measured temperature does not match the set substrate temperature, as shown in Supporting Information Figure 3. On the other hand, the fact that the substrate temperature can be precisely controlled allows us to calibrate the response of the system, as shown in Supporting Information Figure 3. We proceed to correct the measured temperature, without plasma, so that it matches the substrate temperature, and then we use the same correction for the measurements performed under plasma exposure. Figure 3a suggests that the vibrational temperature of PPA adsorbed on alumina significantly increases by ∼80 °C with exposure to even low-powered argon plasma (0.5 W). The actual power to maintain the plasma is calculated through measurements of the RF voltage, the current, and the phase difference between them. Our measurements suggest that approximately 10% of the power supplied by the RF power source is transferred to the discharge, which is reasonable for this type of small plasma reactor in the low power regime (<10 W).^26^ Details of the actual power measurements are summarized in our previous report.^27^ The temperature of PPA increases linearly with the RF input power, as shown in Figure 3b, when the substrate temperature is set to 30 °C. At a plasma power of 0.75 W, the vibrational temperature of PPA increases by ∼110 °C compared to that of the case without argon plasma exposure. At even higher plasma input powers we observe a loss of Raman signal. FTIR measurements, to be discussed later in the letter, suggest that this is due to a combination of desorption from the alumina surface and decomposition of the PPA molecule.

**Figure 3:**
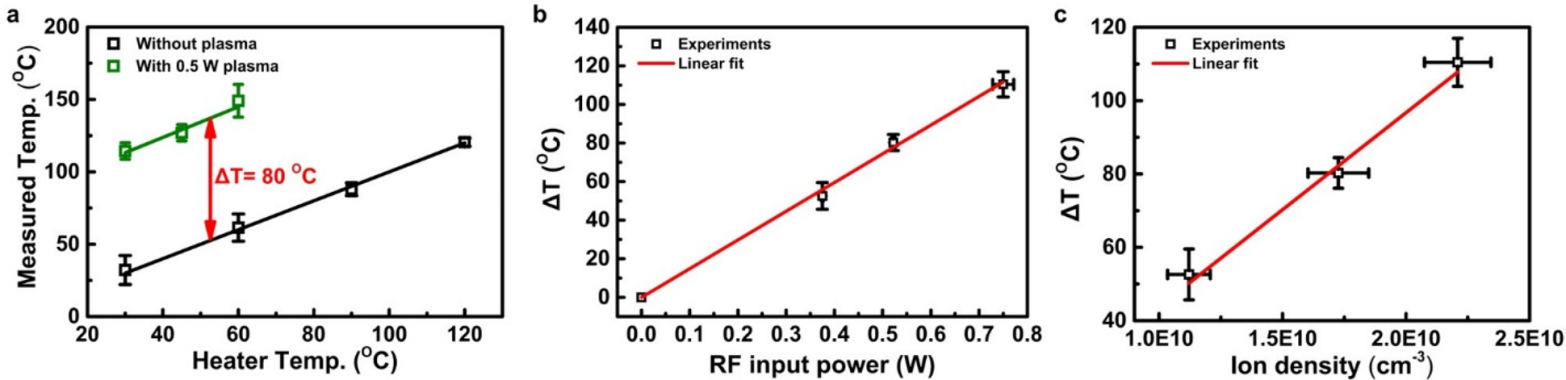
(a) Temperature obtained by Raman spectroscopy as a function of substrate temperature. An 80 °C increase in temperature of PPA with 0.5 W argon plasma exposure is observed. Temperature of PPA (b) as a function of the RF input power and (c) as a function of plasma density. All measurements were repeated three times to determine the error bars. Δ*T* represents additional heating to PPA through argon plasma exposure (i.e., *T*_Raman_ – *T*_Heater_).

We have performed capacitive probe measurements to obtain the ion density, which for the case of a simple quasi-neutral argon plasma corresponds to the plasma density, with the goal of correlating the measured temperature of PPA through Raman thermometry to a readily measurable plasma parameter.^28^ We stress that the relation between plasma density and RF input power strongly depends on reactor design, making it difficult to compare experimental conditions when RF input power alone is provided. Supporting Information Figure 4 shows the measured plasma density as a function of RF input power. We find that the temperature of PPA has a nearly linear relationship with the ion density, as shown in Figure 3c. The plasma density generally increases with the measured RF input power, resulting in an increase rate of energy transfer to plasma-exposed surfaces. The plasma exposure therefore could offer a unique pathway for heating adsorbates on catalysts in a different manner from any other conventional process.^14,29^ It should be mentioned that multiple phenomena may contribute to this surface-targeted heating effect. Plasma-facing surfaces are exposed to fluxes of electrons, ions, and various electronically excited species. Their recombination releases a significant amount of energy (15.76 eV for the case of an electron–ion recombination event, corresponding to the ionization potential of argon). In addition, significant energy is transferred to the surface because of ion acceleration in the electrical double layer, also known as the plasma sheath, formed in front of plasma-facing surfaces. The measured plasma density is therefore a useful parameter, although its measurement alone does not allow us to elucidate which mechanism is primarily responsible for the increase in surface vibrational temperature. More sophisticated experiments have yet to be designed to address this long-standing issue.

We carried out in situ temperature-dependent FTIR measurements to investigate how the plasma irradiation could affect various stretching modes of PPA on alumina and provide additional confirmation of the Raman data. The plasma reactor mounted on the chamber is identical to the one that we used for the Raman thermometry. Experimental conditions, such as the flow rate of argon and pressure, are also the same as mentioned earlier. We keep using the same SERS substrates to adsorb PPA on alumina and set the ramp rate to 10 °C/min through a temperature controller located right below the substrate. Supporting Information Figure 5 presents the FTIR spectrum of PPA on alumina, showing the vibrational frequency stretches for the C–C–C (aromatic benzene ring, 1440 cm^–1^), P=O (1320–1140 cm^–1^), P–O–Al (1152 cm^–1^), P–OH (951–927 cm^–1^), and P–C bonds (754–634 cm^–1^).^30^ For consistency with the Raman study, we investigate the peak at 1440 cm^–1^ corresponding to the aromatic ring of PPA.^20^ We compare the temperature, *T*_max_, at which the peak for the aromatic ring is reduced the most in the absence and presence of argon plasma. Supporting Information Figure 6 describes how we derive the *T*_max_ through the temperature-dependent FTIR measurements.

The binding energy (BE) between PPA and alumina is dependent on the number of bonds involved in their chemisorption. The Redhead analysis has been widely employed to calculate BE between catalysts and adsorbates through *T*_max_ from temperature-programmed desorption experiments.^31^ In this analysis, although a commonly utilized pre-exponential factor is ν = 10^13^ s^–1^, it is particularly suitable for small molecules such as CO. Fichthorn et al. reported an increase in pre-exponential factors with a longer alkane group chain, suggesting that pre-exponential factors could be dependent on the size of molecules.^32,33^ Therefore, applying ν = 10^17^ s^–1^ and *T*_max_ = 186 °C (the case without plasma exposure) to the Redhead equation, the calculated BE is 1.72 eV. Bauer et al. computed the BE between phosphoric acid (PA) and alumina according to the number of bonds and reported BE being 1.69 eV for bidentate bonds. The authors also reported that bidentate bonds are the most stable chemisorption when the PA is exposed to liquid water.^34^ It implies that PPA adsorbs on the surface of alumina, forming bidentate bonds, because PPA was dissolved in DI water before drop-casting in this study. (Details of the sample preparation can be found in the Supporting Information.) Figure 4a suggests that the adsorbed PPA is fully desorbed at 200 °C without any plasma exposure. This result also confirms that the sample likely consists of a monolayer (or less) of PPA since a multilayer is not stable at a temperature of 200 °C. Yagyu et al. find that a multilayer of PPA on alumina is converted to a monolayer by the time the temperature reaches 400 K.^30^ The same is observed for the case of PPA on TiO_2_.^35^ Interestingly, while the reduction of the peak for the aromatic ring occurs at lower temperature with 0.5 W argon plasma irradiation (see spectra at 150 °C in Figure 4a,b), broad peaks for P=O, P–O–Al, and P–C bonds are still observed at 200 °C. Figure 4 suggests that PPA is thermally desorbed without plasma exposure, but PPA decomposition occurs with argon plasma exposure. As mentioned earlier, the recombination of argon ions at the surface releases significant energy of 15.76 eV, which is more than sufficient to decompose PPA. Biswas et al. performed ab initio molecular dynamics calculations to investigate high-temperature decomposition of diisopropyl methylphosphonate on alumina. The authors pointed out that thermally decomposed phosphorus atoms form new P–O bond at the alumina surface that are stable even at 700 °C.^36^ Kalinovych et al. also investigated the thermal decomposition of PPA and reported that P–C and P–C–O bonds remain on the metal surface (Cu) even at 500 °C.^37^ This implies that decomposed PPA may be strongly chemisorbed on the surface of alumina, resulting in peaks for P=O (∼1270 cm^–1^), P–O–Al (∼1155 cm^–1^), and P–C (754–634 cm^–1^) stretches even at 200 °C (Figure 4b). Overall, the measured reduction in the signal from the aromatic ring at 1440 cm^–1^ with plasma exposure is a likely consequence of both the desorption and decomposition of PPA.

**Figure 4:**
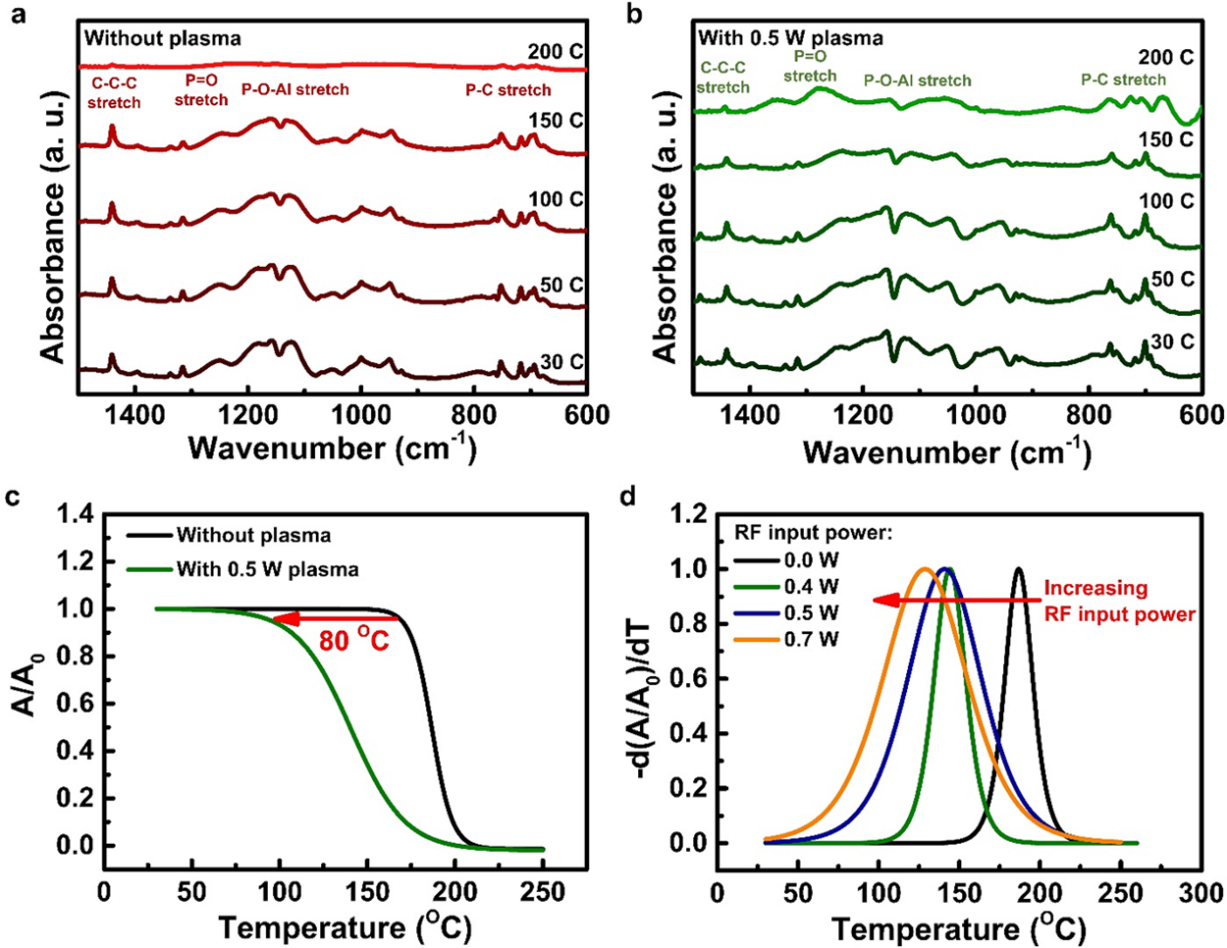
Temperature-dependent IR spectra for PPA (a) without plasma exposure and (b) with 0.5 W argon plasma exposure. (c) Sigmoidal fit of the normalized area (*A*/*A*_0_) obtained by integrating the C–C–C stretching peak at 1440 cm^–1^. (d) Negative derivative of *A*/*A*_0_ with respect to temperature at increasing RF input power.

Interestingly, while exposing the PPA to a 0.5 W argon plasma, the peak corresponding to the aromatic ring starts to decrease in intensity at ∼80 °C lower temperature compared to the case without plasma exposure (Figure 4c). This is consistent with our observations through Raman thermometry, where there is an ∼80 °C increase in the temperature with 0.5 W argon plasma exposure. Figure 4d presents a negative derivative of the sigmoidal fit for the normalized area as a function of temperature. It suggests that the *T*_max_ decreases with increasing RF input power, which is also consistent with the Raman thermometry where the vibrational temperature of PPA increases with increasing RF input power.

In conclusion, we have employed surface-enhanced Raman spectroscopy to quantitatively determine the degree of surface-localized heating for the case of a molecular monolayer under exposure to a low-temperature plasma. We observe a significant increase in the surface vibrational temperature (∼80 °C) even at a relatively low plasma density. The Raman data are confirmed by temperature-dependent FTIR measurements, which show an earlier onset of the loss of signal from the PPA aromatic ring. The increase in surface vibrations correlates linearly with the plasma density, as measured by using an ion probe. The low-temperature plasma leads to fluxes of multiple species directed to exposed surfaces (electrons, ions, various excited states, and photons). While the Raman-based technique described here cannot differentiate among the contributions of these species, it allows us to quantify the vibrational temperature of adsorbates which results from the thermalization of the plasma-produced species at the surface. This is important for the investigation of a broad range of plasma-related processes, from the activation of heterogeneous chemical reactions at catalyst surfaces to the processing of electronic and other functional materials.

## Supporting Information Available

Details of experimental methods including the fabrication of the SERS substrate, Raman thermometry with argon plasma exposure, plasma density measurements, and FTIR measurements (PDF)
