# Supplementary material for: Using Surface-Enhanced Raman Spectroscopy to Probe Surface-Localized Nonthermal Plasma Activation
Source: J Phys Chem Lett. 2024 Apr 9;15(15):4136–41. doi: 10.1021/acs.jpclett.4c00747 (PMC11033932; doi:10.1021/acs.jpclett.4c00747)
Supplement: Supplementary file 1 — jz4c00747_si_001.pdf [file jz4c00747_si_001.pdf]

Journal of Physical Chemistry Letters

Supplementary Information

Using Surface-enhanced Raman Spectroscopy to Probe Surface-localized Nonthermal Plasma Activation

Minseok Kim[a] and Lorenzo Mangolini[a,b*]

[a]*Department of Mechanical Engineering, University of California, Riverside, Riverside, CA, 92521, United States*

[b]*Materials Science & Engineering Program, University of California, Riverside, Riverside, CA 92521, United States*

[*]Corresponding author: Lorenzo Mangolini (lmangolini@engr.ucr.edu)

Methods

Fabrication of Surface Enhanced Raman Spectroscopy (SERS) substrate. For surface cleaning, silicon wafers were sonicated in Acetone for 15 minutes, rinsed three times with acetone and deionized (DI) water, and dried through a nitrogen gun. 3 nm-thick Ag films were deposited onto the cleaned silicon wafers at a rate of 0.1 Å/sec using an E-beam Evaporator (BJD 1800, Airco Temescal). The substrates were then dewetted at 300 ℃ with a flow of 100 sccm of Ar gas in a electrical furnace to make an island-structured Ag (See Figure 1d). The dewetted Ag surfaces were coated with 2 nm of Al_2O_3 at 200 ℃ using an atomic layer deposition system (Savannah 100, Cambridge Nanotech) to adsorb Phenylphosphonic Acid (PPA, P28808, Sigma-Aldrich). PPA was dissolved in DI water with a concentration of 0.01 mol%. The solution was drop-casted onto the substrates, and any remaining DI water and excessive PPA were eliminated at 3.7 torr with a 20 sccm flow of argon in the chamber. Supplementary Figure 1 presents stable chemisorption of PPA onto alumina even after a hour.

Raman thermometry with argon plasma exposure. Figure 1a shows a schematic of the optical system to measure Raman scattering signal from PPA adsorbed on the lab-made SERS substrates. A continuous wave laser at λ=532 nm was irradiated to the sample inside the chamber. Power of laser was controlled by the density filters (Supplementary Figure 3). Both Raman and Rayleigh scattering were collimated by a lens (focal distance = 5 cm) and focused to a monochromator (Acton series, Princeton Instruments). A double notch filter was positioned ahead of the monochromator slit to minimize the Rayleigh scattering signal from the sample. Spectra, taken with a 1 second of acquisition time, were averaged (10 spectra in total) using a software (LightField, Princeton Instruments). This method is well described in our previous study.[1]

Argon plasma exposure was performed using a commercial environmental chamber (HVC-DRP-5 Harrick Scientific). The chamber was modified by replacing one of the KBr windows with

Pyrex tubular reactor (See Figure 1b), allowing the irradiation of plasmas onto the sample. A copper electrode wrapped around the reactor was connected to an RF power supply (RFPP RF-5S, Advanced Energy) and a matching network (MFJ-989D, MFJ) to ignite plasmas. A flow of 20 sccm of argon gas was flowed into the system, controlled by mass flow controllers (MKS 1179C, MKS Instruments). The pressure was maintained at 3.7 torr. The temperature of the system was regulated using a temperature controller (ATK-024- 3, Harrick Scientific).

The actual power to sustain plasmas was obtained by measuring the RF voltage, current, and phase difference during the discharge using an oscilloscope (TES2024C, Tektronix).[2] It is observed that approximately 10% of the power supplied by the RF power source is effectively coupled to the discharge, consistent with our previous experiences with similar small plasma reactors at low power regime.

Plasma density measurements. We performed capacitive probe measurements to obtain the plasma density (i.e., ion density) of argon plasma as a function of RF input power. A copper wire tip with a diameter of 2.5 mm and a length of 0.5 mm was inserted into the chamber by replacing one of the KBr windows. The probe tip was then connected to a 200 pF capacitor and an RF power supply (RFPP RF-5S, Advanced Energy) capable of generating square wave functions for pulsed operation. The capacitor voltage was measured using an oscilloscope (TES2024C, Tektronix). The capacitor was charged negatively through pulses generated by the RF power supply, then discharged by the ion flux from the argon plasma toward the probe.[3]

It was assumed that Ar ions penetrate the sheath edge at the Bohm velocity. It is necessary to know the electron temperature of argon plasma for calculating the Bohm velocity. The electron temperature was estimated by balancing the rate of ionization and ion wall losses.[4] This approach is well summarized in our previous report.[5] We obtained a value of 5.5 eV for the electron temperature and used that value to calculate the plasma density. Supplementary Figure 4 shows the measured ion density as a function of RF input power.

FTIR measurements. The same setup used for Raman measurements has been employed for the FTIR measurements. The reaction chamber (HVC-DRP-5 Harrick Scientific) was mounted on a Praying Mantis diffuse reflectance adapter (DRP-XXX, Harrick Scientific). The adapter was then equipped in a FTIR spectrometer (iS50, Thermo Fisher Scientific). All spectra were obtained through liquid-nitrogen-cooled HgCdTe detector and averaged via 16 scans at a resolution of 4 cm^{-1}. The experimental conditions for plasma exposure are also exactly the same as those used for the Raman measurements for the consistency. We set the ramp rate at 10 ℃/min through the temperature controller (ATK-024- 3, Harrick Scientific).

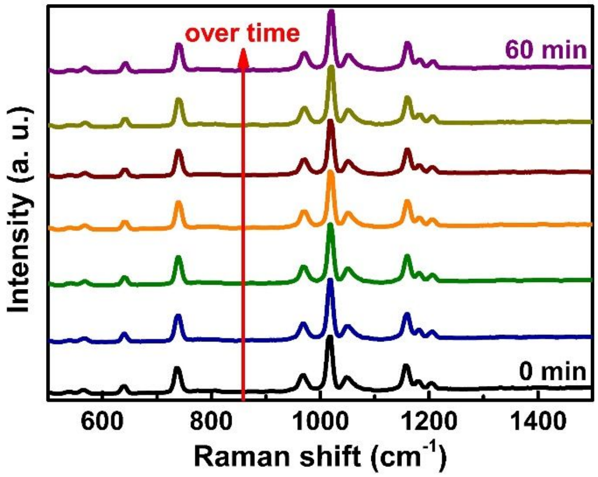

Supplementary Figure 1. Stable Raman signature for Phenylphosphonic Acid over 1 hour.

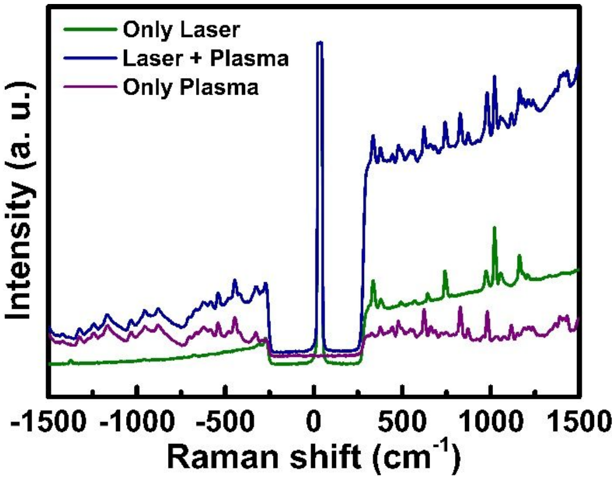

Supplementary Figure 2. Raman spectrum with only laser (black line), laser with argon plasma (red line), and only argon plasma (blue line).

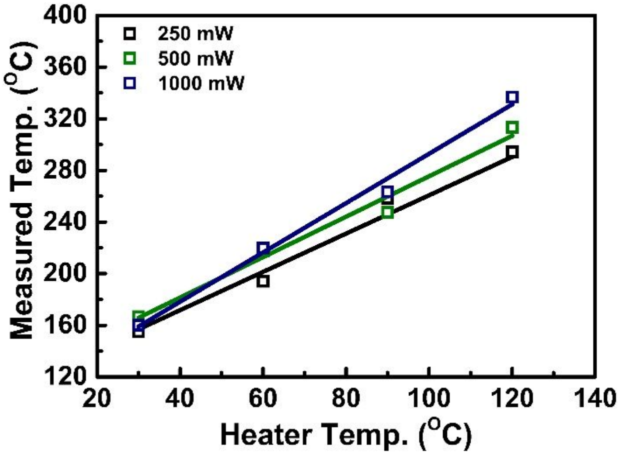

Supplementary Figure 3. Temperature of Phenylphosphonic acid measured via Raman spectroscopy as a function of heater temperature, while varying the power of the laser (without plasma exposure).

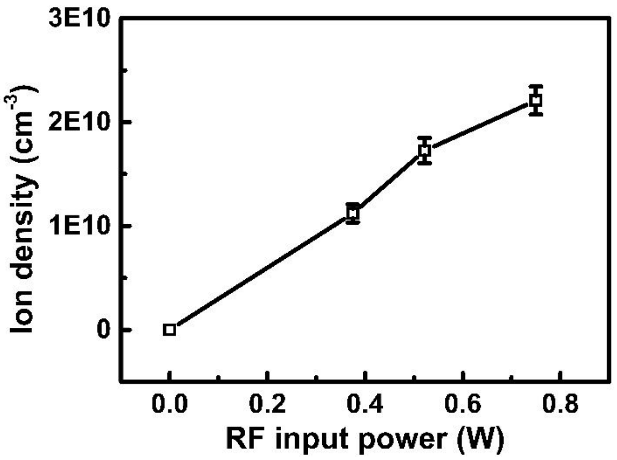

Supplementary Figure 4. Measured ion density as a function of RF input power.

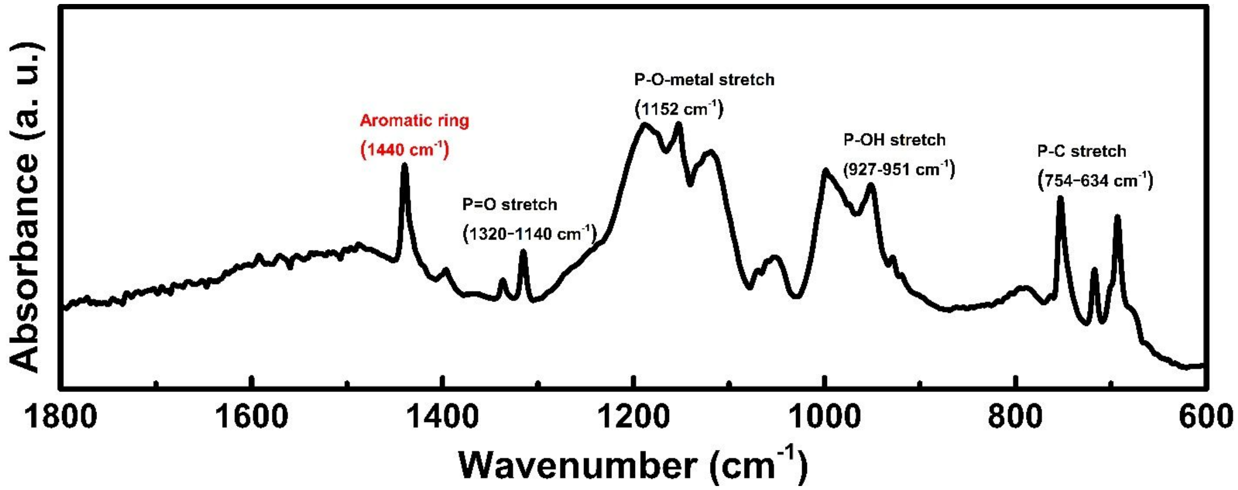

Supplementary Figure 5. FTIR spectrum of phenylphosphonic acid on alumina.

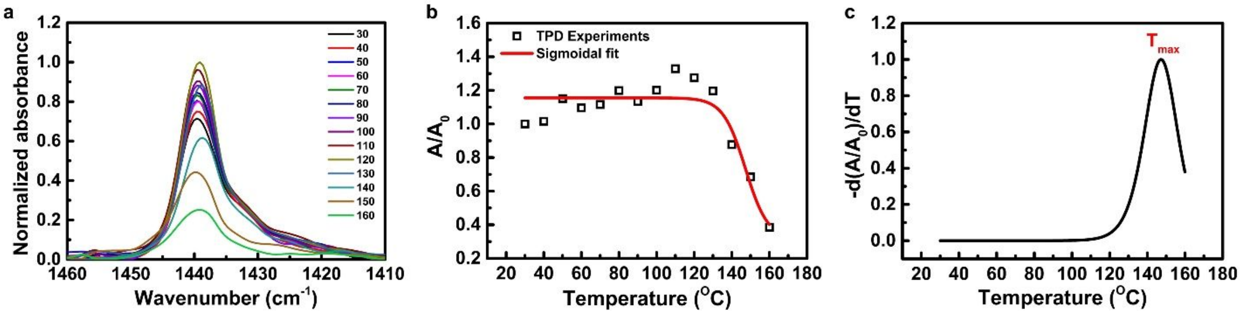

Supplementary Figure 6. (a) The FTIR spectrum of PPA on alumina at 1 min interval, as increasing temperature. (b) The normalized area (A/A_0) obtained by integrating each spectrum along with a sigmoidal fit. (c) The negative derivative of the sigmoidal fit.

References

(1) Berrospe-Rodriguez, C.; Schwan, J.; Nava, G.; Kargar, F.; Balandin, A. A.; Mangolini, L. Interaction between a Low-Temperature Plasma and Graphene: An in Situ Raman Thermometry Study. *Phys. Rev. Appl.* **2021**, *15*, 024018.

(2) Godyak, V. A.; Piejak, R. B. In situ Simultaneous Radio Frequency Discharge Power Measurements . *J. Vac. Sci. Technol. A* **1990**, *8*, 3833–3837.

(3) Braithwaite, N. S. J.; Booth, J. P.; Cunge, G. A Novel Electrostatic Probe Method for Ion Flux Measurements. *Plasma Sources Sci. Technol.* **1996**, 5, 677–684.

(4) Sode, M.; Jacob, W.; Schwarz-Selinger, T.; Kersten, H. Measurement and modeling of neutral, radical, and ion densities in H_2-N_2-Ar plasmas. *J. Appl. Phys.* **2015**, 117, 083303.

(5) Kim, M.; Biswas, S.; Nava, G.; Wong, B. M.; Mangolini, L. Reduced Energy Cost of Ammonia Synthesis Via RF Plasma Pulsing. *ACS Sustain. Chem. Eng.* **2022**, *10*, 15135–15147.
